# Editorial

**Published:** 1914-10

**Authors:** 


### EDITORIAL

The Business Office of the *Journal-Record* is 23 West Alabama Street.

The Editorial Office is 1014-15 Atlanta National Bank Building.

Address all Business Communications to *Journal-Record of Medicine*, 23 West Alabama Street.

Make remittances payable to *The Journal-Record of Medicine*.

On matters pertaining to the Editorial and Original Communications, address Edgar G. Ballenger, M. D., Atlanta, Ga.

Reprints of Original Articles will be furnished at cost price. Requests foi the same should always be made in THE MANUSCRIPT.

We will present, postpaid, on request to each contributor of an original article, twenty (20) marked copies of *The Journal-Record of Medicine* containing such article.

---

### ASSOCIATED CHARITIES.

In these days when we are being called upon by every newspaper to consider the sorrows and needs of our fellow men all over the world, when distress and perturbation of mind and reason create still further trouble of the same sort by leading men to useless and awful warfare, when our attention is taken to deplorable conditions far away from our doors, we must not forget what it is shill demanded within our household and that we have many objects for our solicitude and care.

The poor are always with us, but the methods of caring for them are steadily changing, from day to day as we grow and develop and as the problems receive more and more skilled attention.

Our civilization demands that we do for others many times what they can not do for themselves. This modification of the golden rule has been accepted and in its application, we have learned that in the long run it is profitable and that we are doing for ourselves.

Every normal person knows that there is a satisfaction in doing some philanthropic work. A man feels better when he has helped some one in distress of mind or body whether there is any other profit in it or not. What he really wants to be assured of when he makes a sacrifice of his time or his means however small or large, is that the gift has been well applied and will result in the greatest good.

In Atlanta we have for many years been giving our aid through the Associated Charities and have felt secure that through the able and conscientious management of this organization the means of relief reached their objects in the best way. But somehow, we have come to believe that the organization was a thing of itself and could now get along without us. Thus it has happened that it has been about to close its offices and deprive its beneficiaries of the assistance they need because of lack of subscriptions.

This was announced some little time ago, but even then the citizens did not realize what it meant and each rather left it to some other to do something about it.

When the newspapers announced what seemed to be the final closure of the offices and the disbanding of the forces, we awakened to the fact that here at home in Atlanta there was as much for us to do as could possibly demand attention from us concerning the world at large.

The Associated Charities comprises a Board of Managers, a Secretary in charge and a corps of trained workers. Offices are needed and funds for the payment of salaries as well as for buying the materials of relief.

The administration during the growth of the institution has been excellent and has kept pace with the times. Indigent people have been relieved, idle have been given work, children have been rescued and placed under proper care, discouraged families have been prevented from rupture or reunited, the sick have been given attention and medicine, and innumerable other troubles have been relieved unostentatiously and successfully. Not only has the association been of value to the people it cared for, but it has opened the way for individuals or groups to do the right thing when they have been applied to for help. In these days there are many men going about seeking sustenance from the busy people to whom an appeal, coupled with a shabby appearance, means a response of some kind. But no one wants to feel that he is being imposed upon and men have come to understand that the Associated Charities knows how to investigate such matters and to do what is best all around.

We are glad to state that there has been a largely attended and enthusiastic meeting of the citizens and that the Atlanta spirit has been awakened to the needs of this philanthropy. We hope that the Association will go on and have all the support it requires. It is not entirely a local matter because many of the applicants for assistance come from all over the State as well as from neighboring States. Atlanta is large enough to be a Mecca for pilgrims all over the South who come seeking its blessings. We are ready to give them, but oft times the seekers fail because of their own frailties. Even to these we must continue to give help and in all ways make the world better and happier. The Associated Charities give to Atlantans and all interested people the best way of dispensing charity where it is needed fully as much as in the battle-scarred countries that startle us with their unusual and harrowing pictures.

We hope the Profession will do its share in maintaining this organization. R. R. D.

